# Importance of Heat and Pressure for Solubilization of Recombinant Spider Silk Proteins in Aqueous Solution

**DOI:** 10.3390/ijms17111955

**Published:** 2016-11-23

**Authors:** Justin A. Jones, Thomas I. Harris, Paula F. Oliveira, Brianne E. Bell, Abdulrahman Alhabib, Randolph V. Lewis

**Affiliations:** 1 Department of Biology, Utah State University, Logan, UT 84341, USA; justin.a.jones@usu.edu (J.A.J.); paula.oliveira@usu.edu (P.F.O.); brianne.bell@aggiemail.usu.edu (B.E.B.); abood.alhabib@hotmail.com (A.A.); 2 Department of Biological Engineering, Utah State University, Logan, UT 84341, USA; tiharristh@gmail.com

**Keywords:** recombinant spider silk proteins, fibers, aqueous, solvation, heat, pressure

## Abstract

The production of recombinant spider silk proteins continues to be a key area of interest for a number of research groups. Several key obstacles exist in their production as well as in their formulation into useable products. The original reported method to solubilize recombinant spider silk proteins (rSSp) in an aqueous solution involved using microwaves to quickly generate heat and pressure inside of a sealed vial containing rSSp and water. Fibers produced from this system are remarkable in their mechanical ability and demonstrate the ability to be stretched and recover 100 times. The microwave method dissolves the rSSPs with dissolution time increasing with higher molecular weight constructs, increasing concentration of rSSPs, protein type, and salt concentration. It has proven successful in solvating a number of different rSSPs including native-like sequences (MaSp1, MaSp2, piriform, and aggregate) as well as chimeric sequences (FlAS) in varied concentrations that have been spun into fibers and formed into films, foams, sponges, gels, coatings, macro and micro spheres and adhesives. The system is effective but inherently unpredictable and difficult to control. Provided that the materials that can be generated from this method of dissolution are impressive, an alternative means of applying heat and pressure that is controllable and predictable has been developed. Results indicate that there are combinations of heat and pressure (135 °C and 140 psi) that result in maximal dissolution without degrading the recombinant MaSp2 protein tested, and that heat and pressure are the key elements to the method of dissolution.

## 1. Introduction

Recombinant spider silk proteins are of great interest to replace petroleum derived fibers and materials. Native spider silk fibers’ combination of tenacity and strength make it an ideal material to replace common threads such as nylon. Since spiders cannot be farmed like silkworms, spider silk proteins have to be produced synthetically by organisms and methods that do not, and are not capable of duplicating the methodology that a spider uses to produce the silk proteins and maintain their solubility until needed. Spiders have evolved a complex production and storage system to produce large proteins (250+ kDa), transport them to the lumen of the gland, and maintain them in a soluble form until needed [1,2,3].

In order for the spiders to produce a fiber with such remarkable properties, they evolved protein sequences that are some of the most highly repetitive identified to date [4]. These repetitive portions of the protein are what gives a spider’s silk the robust, and varied, mechanical properties [5,6,7,8,9,10]. The proportion of helical to crystalline structures in the sequence and resulting fiber is largely what determines the degree of extensibility vs. ultimate tensile strength in native silk fibers [7,11,12]. Prime examples of this proportion are flagelliform and minor ampullate spider silks. Flagelliform is comprised of helical structure sequences (GPGGX/GPGQQ and GGX) that result in either an elastic spiral or 3_10_-helix, and it lacks the sequences (poly-GA and poly-A) that form β-sheets in the fiber [11]. As a result, flagelliform has 200+% elasticity. Minor ampullate is largely comprised of the poly-GA sequences that form β-sheets in the fiber and GGX helices, but has no GPGGX sequences, resulting in only 5% extension [11,13,14,15].

The non-repetitive amino and carboxyl termini of the proteins are highly conserved and their primary role is production of the protein and its maintenance inside the lumen of the gland in a stable, soluble form [3,16]. The amino termini contain a secretion signal while the carboxyl terminal functions to maintain solubility through the formation of micellar like structures in the lumen of the gland [1,2,3,17]. The carboxyl terminal has been shown to function as a molecular switch in the conversion of the protein from liquid crystal to solid fiber while also functioning to align protein molecules within the fiber [18]. 

Recombinant production of spider silk proteins (rSSPs) has been explored in a variety of host organisms including yeast, plants, goats, and bacteria [19,20,21,22,23,24]. These recombinant systems are all dissimilar to spiders and result in a variety of problems including: truncated proteins, protein aggregation, poor production, and reduced protein size when compared to native sequences. Generally speaking, because host systems are ill-equipped to produce native sized rSSPs and cannot mimic the production and storage of a spider, the sequences have been shortened by reducing the number of repeats and/or eliminating the amino or carboxyl sequences [19,20,21,22,23,24,25,26,27]. While investigators are designing and producing more native length proteins, the trade-off is reduced production and solubility with increased truncation [28]. While these production systems can produce soluble forms of the rSSPs, high level expression can induce precipitation as well, as during the purification process, as the rSSPs becomes less dilute and more pure, the rSSPs both spontaneously self-assemble and precipitate or, are salted out of solution as part of that process [25,29,30]. The purified rSSPs can be lyophilized for storage, generally further reducing the aqueous solubility [29,31]. Necessary to forming fibers, and other materials, is that the rSSPs must be dissolved.

A spider’s web does not dissolve in humid or rainy conditions out of necessity. The loss of the web and energy required to rebuild it would be an evolutionary disadvantage. As such, when rSSPs are produced from these same sequences and precipitated, β-sheets form much like in the fiber and the rSSPs are largely water insoluble [31]. Due to this solubility issue, the majority of rSSPs have been solubilized using 1,1,1,3,3,3 Hexafluoroisopropanol (HFIP) [25,26,27,28,29,32,33]. While HFIP is effective, solubilizing both native spider silk as well as rSSPs, it is not ideal from cost or environmental perspectives. Further, spiders spin silk from aqueous solutions, suggesting that rSSPs can be solvated similarly, without the use of harsh organic solvents. Several methods have been developed that dissolve rSSPs in aqueous solutions.

**Figure 1:**
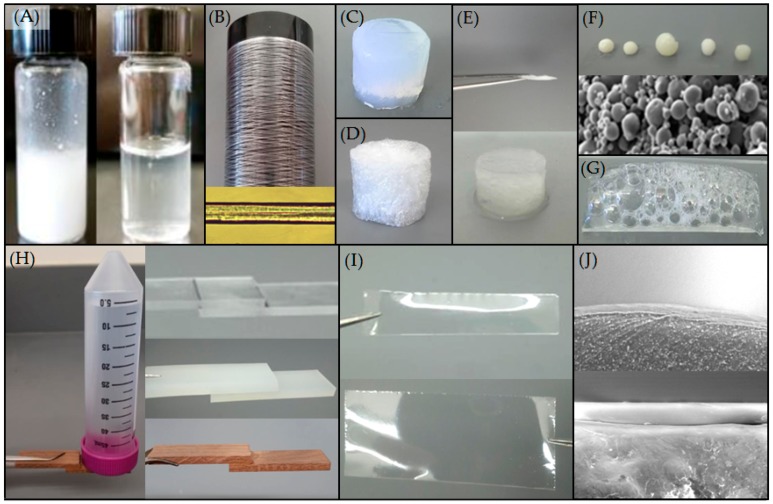
(**A**) **Left**: Suspension of 3% *w*/*v* rMaSp1; **Right**: Same as left solvated by microwave method in approximately 30 s; (**B**) **Top**: Fibers spun from solvated 12% *w*/*v* 80:20 rMaSp1:rMaSp2; **Bottom**: Microscopic image of single fiber at 400× magnification; (**C**) Hydrogel from solvated 6% *w*/*v* 50:50 rMaSp1:rMaSp2; (**D**) Aerogel derived from 6% *w*/*v* 50:50 rMaSp1:rMaSp2 hydrogel; (**E**) Sponge made from solvated 6% *w*/*v* 50:50 rMaSp1:rMaSp2. **Top**: Sponge compressed completely flat before absorbing liquid; **Bottom**: Sponge after absorbing water; (**F**) **Top**: Macrospheres made from recombinant spider silk by dripping solubilized rMaSp1 into liquid nitrogen; **Bottom**: SEM micrographs of rMaSp1 microspheres; (**G**) Foam produced from 6% *w*/*v* 80:20 MaSp1:MaSp2; (**H**) Adhesives, from solvated 12% *w*/*v* 50:50 rMaSp1:rMaSp2, gluing different materials together. **Left** and **bottom right**: wood substrates; **Top right**: polycarbonate substrate; **Center right**: silicone substrate; (**I**) Thin films, 20–30 µm, made from solvated 6% *w*/*v* 80:20 rMaSp1:rMaSp2 spread in thin molds; (**J**) SEM micrographs of recombinant spider silk coatings on silicone and polycarbonate with thicknesses of 5–10 µm.

An inclusive review of current aqueous solvation methods has been published recently [34]. To summarize other methodologies, the authors would argue there are four groupings in the literature. The first is to dissolve the rSSPs in chaotropic solutions, slowly remove the chaotropic reagents, and then concentrate to form the dope [23]. Secondly, in some cases, the natural ability of the rSSPs to self-assemble can be utilized to form concentrated spin dopes. In one reported case, the purified rSSPs proteins naturally self-assembled when purified and concentrated during elution from Ni^++^ chromatography. Fibers either formed as the rSSPs dripped from the end of the chromatography column or accumulated as oily-like films on eluted, purified fractions from which fibers were pulled [32]. Thirdly, rSSPs have again been solubilized with strong chaotropic reagents, then dialyzed into pH 8.0 buffered sodium chloride where the rSSPs segregate into low and high density phases creating a dope solution [35]. Thirdly, high concentration solutions of rSSPs were directly obtained by dissolving rSSPs in sodium thiocyanate and acetate or lithium chloride and formic acid. From these high concentration solutions, fibers were obtained from traditional wet-spinning as well as electrospinning, and thin films formed [36]. Finally, direct solvation of the rSSPs at high concentration is achieved via a method developed in the authors’ laboratory [31]. Recombinant spider silk proteins are sealed inside a glass vial, or other sealed microwave safe container, with water alone without other additives. The solution is microwaved in five second bursts which rapidly generates heat, and thus pressure, inside the sealed vial (Figure 1A). The 2–3 mL of rSSPs is generally solvated within 30 total seconds in the microwave, with larger constructs requiring greater time and energy to solvate. From this method fibers, films, gels, foams, sponges, micro and nanospheres, adhesives, and coatings have been produced (Figure 1B–J), all from the same rSSPs at varying concentrations [29,31]. This article is designed to further describe the microwave method reported by our group as several key aspects of rSSPs solvation via this method have been further investigated and are reported below. Additionally, an alternative means of applying heat and pressure has been developed and characterized for rMaSp2 that is substantially more precise.

## 2. Diversity of Proteins and Fibers

To date, rSSPs from both goats and bacteria have been solvated with the microwave method. Goat derived proteins are rMaSp1 and rMaSp2 fragments of roughly 65 kDa with the highly conserved, non-repetitive carboxyl terminus [29,31]. These rSSPs have been solvated from a lyophilized state as well as wet protein mass. Protein solutions of 40% *w*/*v* rMaSp1 have been achieved with this method. Generally speaking, a suspension of 80:20 rMaSp1:rMaSp2 at 15% *w*/*v* can be solvated in under 30 s in the microwave in water only. Interestingly, solutions containing rMaSp2 tend to form hydrogels with gelation times decreasing with increasing concentrations of rMaSp2. While rMaSp1 solutions can be maintained on the bench (10%–15% *w*/*v*) for days, inclusion of rMaSp2 markedly decreases time to gelation. Solutions of rMaSp2 require roughly twice the energy input of rMaSp1 or chimeric sequences such as FlAS_3_ and A_4_S_8_ or a piriform analogue [4,8,32] (Table 1). These proteins have ranged from 60 kDa to 180 kDa and the majority have been solvated from a lyophilized state in the 5%–30% *w*/*v* range. As would be suspected, the amount of microwave energy required, and thus heat and pressure, to solvate rSSPs increases as the rSSPs increase in molecular weight, with some constructs requiring more energy (MaSp2) than others (MaSp1 and chimeric sequences) for reasons that are not completely understood at this point.

**Figure 2:**
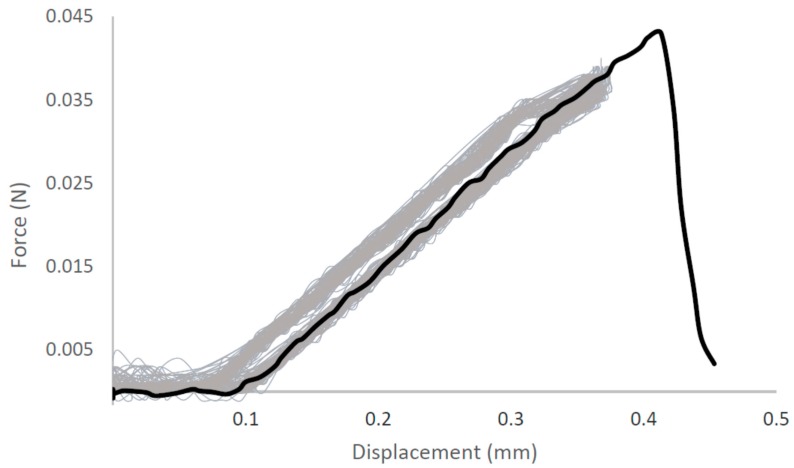
A 19 mm fiber spun from aqueous solution repeatedly cycled 99 times at 5 mm/min to 80% maximum load and then tested to failure.

Despite this variety of sequences and sources of rSSPs they all can form fibers, films, gels, adhesives, and macro as well as micro particles and coatings when solvated using the microwave method. Spinning fibers from HFIP dopes has been developed for a number of years and their mechanical properties are remarkable [23,37]. Spinning fibers from aqueous solutions is a very recent advancement, but the mechanical properties from the same proteins using the same dual stretch extrusion system are similar [31,38]. Other rSSPs fibers reported in the literature have increased extension at the expense of stress [25,28,32,38]. While this produces fibers with an energy to break similar to native silks, the mechanical properties themselves are not similar. Utilizing a programmable, mechanical wet-spinning instrument, fiber properties from this microwave based aqueous method more closely resemble those of native silks [31]. Of note, fibers are now being spun from aqueous solutions that can be cyclically loaded and maintain their mechanical properties (Figure 2). Repeated cyclic loading of a fiber spun from 10% *w*/*v* aqueous solution of an 80:20 blend of rMaSp1:rMaSp2 to 80% maximum stress demonstrates that fibers spun from this aqueous method and spinning system more closely resemble what has been reported in the literature for natural spider silks [9]. Only minor fiber deformation occurs during the initial stretch and the remaining 99 cycles are nearly identical in both stress and strain.

## 3. Salt Influences Solubility

Regardless of the rSSPs tested, one commonality that is becoming increasingly evident is that proteins need to be substantially free of contaminating salts. As has been widely reported, desalting is critical to fiber formation [29,31,38]. Salts interrupt the highly ordered polymer blocks and do not allow complete self-assembly of the rSSPs into fibers or other materials. There are some notable exceptions in some chimeric rSSPs sequences [32]. The water soluble forms of the proteins were not in water alone. Rather, they were diluted with purification buffers which prevented their precipitation. Fibers produced from this method had poor mechanical performance, likely due to salt inclusion in the fiber disrupting the structure. Common to all attempts to spin fibers, or produce materials from rSSPs, is the need for pure protein. Impurities in the form of salts, buffers, or contaminating proteins either diminishes mechanical properties or inhibits the ability to form fibers or other materials.

Salt concentrations have proven critical with this solvation method. When too great a salt concentration is present (>800 µS/cm), solvation of the rSSPs becomes much more challenging by this method. While salts do not completely inhibit solvation, their presence reduces the efficiency of solvation of rSSPs and, depending on the rSSPs and conditions, leaves substantially more rSSPs in suspension. In the original publications, additives to the dopes were used [29,31]. Acids and free amino acids were found to ease the dissolution and also increased gelation times. As the group has become more experienced in the method, and as salts have been more completely removed from the purified protein powders, high salt concentrations were determined to be the reason that acidifying or adding free amino acids were required. As has been reported, decreasing pH as the silk proteins travel down the spider’s duct may be required to accommodate the approximately 180 mM NaCl concentration within a spider’s gland in order to form fibers [39]. In this method, salt concentrations below 800 µS/cm result in near 100% solvation and recovery of rSSPs without altering pH or adding any other substances to the water. 

## 4. Solubilization without Microwaves

Due to the inherent inability to finely control a traditional microwave, further experimentation was performed using a Parr stirred pressure reactor (4848B reactor controller, model A2590HC13EB reactor with heated and cooled aluminum block) to more precisely generate and control heat and pressure parameters and study their effect on the solubilization of rMaSp2 containing the C-term sequence (Table 1). Table 2 outlines the temperatures and pressures achieved as well as the time that it took to accomplish those temperatures and pressures. The Parr vessel was first pressurized with house-air to 90 psi then ramped to the prescribed temperature, and then immediately after achieving the prescribed temperature the internal cooling loop was activated with ice cold water to bring the solution temperature down. There was a minimal (<30 s) delay time once the prescribed temperatures were achieved. All experiments were done with 60 g of wet rMaSp2 protein pellet produced in *E. coli* in 200 mL of DI-H_2_O. This wet protein pellet was purified to this point via ammonium sulfate precipitation and collected using a continuous flow centrifuge. While ramping to temperature, and also while cooling, the contents of the Parr vessel were agitated with an internal mixing impeller set to 600 rpm. This served to not only evenly apply heat but also to effectively disperse the protein pellet into the water.

**Figure 3:**
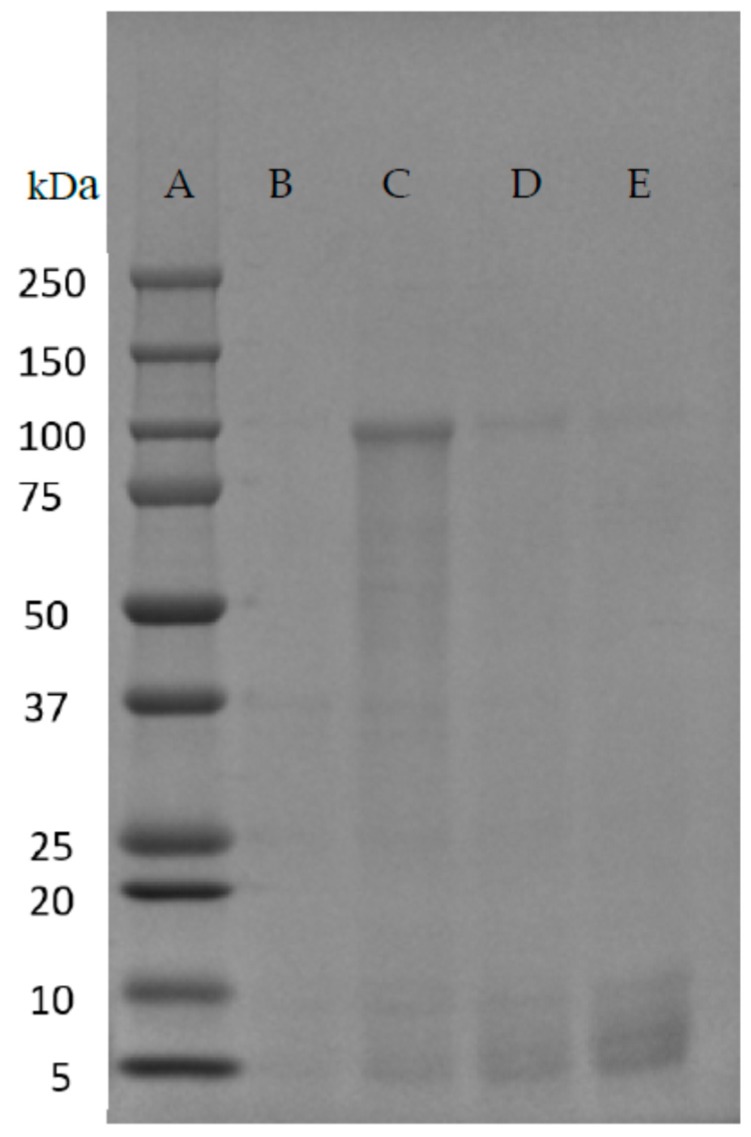
SDS-PAGE of Parr vessel temperature/pressure solubilization of rMaSp2 (predicted 98.7 kDa). (**A**) Bio-Rad Dual Color prestained protein molecular weight (MW) marker (MW listed to the left); (**B**) Soluble rMaSp2 at 120 °C and 127 psi; (**C**) Soluble rMaSp2 at 130 °C and 131 psi; (**D**) Soluble rMaSp2 at 140 °C and 155 psi; (**E**) Soluble rMaSp2 at 150 °C and 161 psi.

The results, when analyzed by SDS-PAGE and coomassie staining, demonstrated that 130 °C and 131 psi were able to solvate the protein without degradation. While 120 °C was too little heat and or pressure to solubilize, temperatures of 140 °C and above led to partially or completely degraded protein (Figure 3).

**Figure 4:**
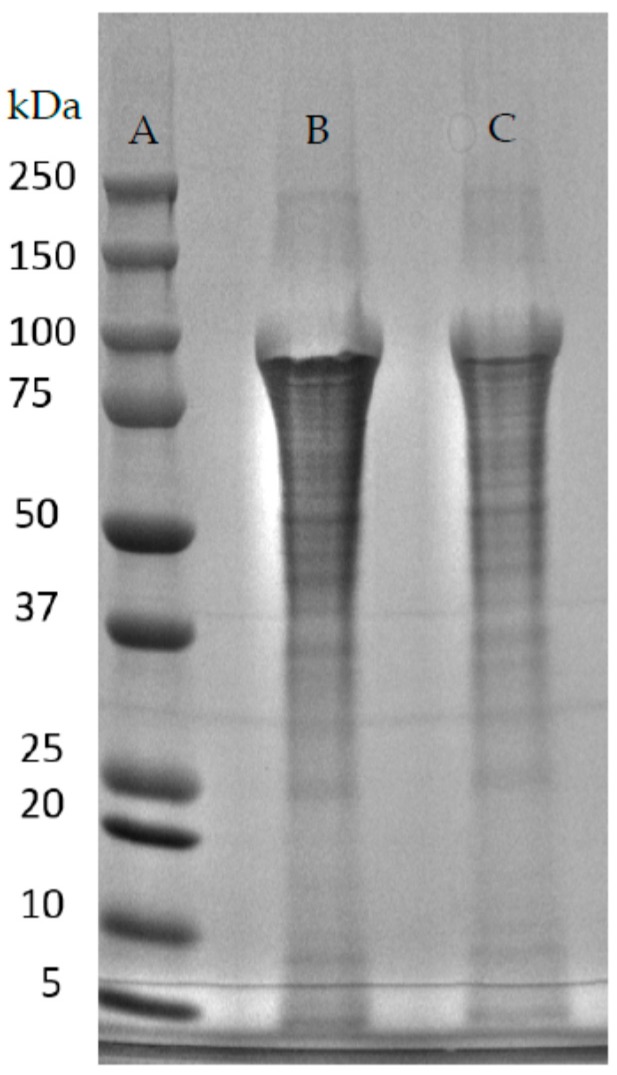
SDS-PAGE of Parr vessel temperature and pressure solubilization of rMaSp2 (predicted 98.7 kDa). (**A**) Bio-Rad Dual Color prestained protein molecular weight marker (MW listed to the left); (**B**) Soluble rMaSp2 at 135 °C and 140 psi; (**C**) Soluble rMaSp2 at 130 °C and 131 psi.

The initial experiment (Figure 3) was done in 10 °C increments with 130 °C and 131 psi discovered as being the most efficient at solubilizing the protein without degradation. A further experiment with the Parr vessel was performed at 135 °C achieving 140 psi in 17 min and compared, in the same experiment with 130 °C and 131 psi (Figure 4). Again, this experiment was carried out with 60 g of wet rMaSp2 protein pellet with C-term sequence. 

The SDS-PAGE with coomassie stain indicates that more protein is solubilized without being degraded at 135 °C and 140 psi when compared to 130 °C and 131 psi. However, SDS-PAGE analysis may be insufficient to determine over all levels of degradation, as some products are potentially small enough to be undetectable via this method.

## 5. Conclusions

The generation of heat and pressure appear to be the key elements to solvating recombinant spider silk proteins in aqueous solution. Whether through the use of a microwave and sealed vial or the controllable Parr vessel, these two elements result in dissolution of the rSSp. Clearly from the Parr vessel experiments, controlling the heat and pressure is important as the protein can be degraded if too high a temperature and pressure are used, which represents a distinct disadvantage of a standard microwave. 

The microwave method of generating heat and pressure is rapid and capable of producing a range of materials from the solvated rSSPs. Fibers as well as films, foams, gels, coatings, adhesives, and macro and microspheres and sponges have been produced from the same starting rSSPs formulations [31,40]. A diverse range of rSSPs with differing protein sequences have been solvated ranging in size from 60 to 180 kDa, with and without the non-repetitive, highly conserved carboxyl terminus, that have been recombinantly produced in goats as well as bacteria (Table 1).

A Parr heated pressure vessel provides an alternative means to solvate rSSPs; in this case rMaSp2 with C-term sequence was used. Specifically for this rMaSp2 construct, 135 °C and 140 psi appears to optimally bring the protein into solution without degradation, while temperatures and pressures above this degrade the protein. Below this temperature, the rMaSp2 protein is either not completely solvated or not solvated at all. Salt concentrations in this system have not been explored as to how they influence solubility of the rSSPs, however, it is unlikely to be substantially different than observations made in the microwave system. This method appears to be much more scalable and controllable than conventional microwaves and can be imagined as a means from which to continuously spin fibers. The inherent pressure of the Parr vessel system could be used to extrude the material after solvation. This method also demonstrates that microwaves are not needed for solubilization and that heat and pressure alone can solvate this rSSp.

It is necessary, but not thought to be sufficient, to have heat and pressure; residual salt from purification of the rSSp needs to be removed prior to solvation. Trials using the microwave method indicate that in order to form fibers or other materials, the conductivity needs to be below 800 µS/cm. Above this point, not only does dissolution of the rSSp become difficult, but materials properties suffer as well. 

## Figures and Tables

**Table 1:** Heterologous spider silk protein sequences.

Native-Like Sequences (Host Organism and Construct)
**C-Term:**
SRLSSPQASSRLSSAVSNLVATGPTNSAALSSTISNVVSQIGASNPGLSGCDVLIQALLEVVSALIQILGSSSIGQVNYGSAGQATQIVGQSVYQALG
**Goat MaSp1 with C-Term:**
QGAGAAAAAAGGAGQGGYGGLGGQGAGQGGYGGLGGQGAGQGAGAAAAAAAGGAGQGGYGGLGSQGAGRGGQGAGAAAAAAGGAGQGGYGGLGSQGAGRGGLGGQGAGAAAAAAAGGAGQGGYGGLGNQGAGRGGQGAAAAAAGGAGQGGYGGLGSQGAGRGGLGGQGAGAAAAAAGGAGQGGYGGLGGQGAGQGGYGGLGSQGAGRGGLGGQGAGAAAAAAAGGAGQGGLGGQGAGQGAGASAAAAGGAGQGGYGGLGSQGAGRGGEGAGAAAAAAGGAGQGGYGGLGGQGAGQGGYGGLGSQGAGRGGLGGQGAGAAAAGGAGQGGLGGQGAGQGAGAAAAAAGGAGQGGYGGLGSQGAGRGGLGGQGAGAVAAAAAGGAGQGGYGGLGSQGAGRGGQGAGAAAAAAGGAGQRGYGGLGNQGAGRGGLGGQGAGAAAAAAAGGAGQGGYGGLGNQGAGRGGQGAAAAAGGAGQGGYGGLGSQGAGRGGQGAGAAAAAAVGAGQEGIRGQGAGQGGYGGLGSQGSGRGGLGGQGAGAAAAAAGGAGQGGLGGQGAGQGAGAAAAAAGGVRQGGYGGLGSQGAGRGGQGAGAAAAAAGGAGQGGYGGLGGQGVGRGGLGGQGAGAAAAGGAGQGGYGGVGSGASAASAAASRLSSPQASSRLSSAVSNLVATGPTNSAALSSTISNVVSQIGASNPGLSGCDVLIQALLEVVSALIQILGSSSIGQVNYGSAGQATQIVGQSVYQALG
**Goat MaSp2 with C-term:**
PGGYGPGQQGPGGYGPGQQGPSGPGSAAAAAAAAAAGPGGYGPGQQGPGGYGPGQQGPGRYGPGQQGPSGPGSAAAAAAGSGQQGPGGYGPRQQGPGGYGQGQQGPSGPGSAAAASAAASAESGQQGPGGYGPGQQGPGGYGPGQQGPGGYGPGQQGPSGPGSAAAAAAAASGPGQQGPGGYGPGQQGPGGYGPGQQGPSGPGSAAAAAAAASGPGQQGPGGYGPGQQGPGGYGPGQQGLSGPGSAAAAAAAGPGQQGPGGYGPGQQGPSGPGSAAAAAAAAAGPGGYGPGQQGPGGYGPGQQGPSGAGSAAAAAAAGPGQQGLGGYGPGQQGPGGYGPGQQGPGGYGPGSASAAAAAAGPGQQGPGGYGPGQQGPSGPGSASAAAAAAAAGPGGYGPGQQGPGGYAPGQQGPSGPGSASAAAAAAAAGPGGYGPGQQGPGGYAPGQQGPSGPGSAAAAAAAAAGPGGYGPAQQGPSGPGIAASAASAGPGGYGPAQQGPAGYGPGSAVAASAGAGSAGYGPGSQASAAASRLASPDSGARVASAVSNLVSSGPTSSAALSSVISNAVSQIGASNPGLSGCDVLIQALLEIVSACVTILSSSSIGQVNYGAASQFAQVVGQSVLSAF
***E. coli* MaSp1 with and without C-term:**
(GAGQGGYGGLGSQGAGRGGLGGQGAGAAAAAAAAGGAGQGGYGGLGSQGAGRGGLGGQGAGAAAAAAAAAGQGGYGGLGSQGGGAGQGGYGGLGSQGAGRGGLGGQGAGAAAAAAAAGGAGQGGYGGLGSQGAGRGGLGGQGAGAAAAAAAAGGAGQGGYGGLGSQGAGRGGLGGQGAGAAAAAAAAGGAGQGGYGGLGSQGAGRGGLGGQGAGAAAAAAAAAGQGGYGGLGSQGGGAGQGGYGGLGSQGAGRGGLGGQGAGAAAAAAAAGGAGQGGYGGLGSQGAGRGGLGGQGAGAAAAAAAA)_2_
***E. coli* MaSp2 with and without C-term:**
(GGPGQQGPGGYGPGQQGPSGPGSAAAAAAAAGPGQQGPGGYGPGQQGPGGYGPGQQGPSGPGSAAAAAAAAGPGGYGPGQQGPGGYGPGQQGPGGYGPGQQGPSGPGSAAAAAAAAGPGQQGPGGYGPGQQGPGGYGPGQQGPSGPGSAAAAAAAAGPGQQGPGGYGPGQQGPSGPGSAAAAAAAAGPGQQGPGGYGPGQQGPGGYGPGQQGPSGPGSAAAAAAAAGPGGYGPGQQGPGGYGPGQQGPGGYGPGQQGPSGPGSAAAAAAAAGPGQQGPGGYGPGQQGPGGYGPGQQGPSGPGSAAAAAAAAGPGQQGPGGYGPGQQGPSGPGSAAAAAAAA)_4_
***E. coli* Piriform with and without C-term:**
(VSQVQQASIQQAQSSSAQSRQSSVAQQASISQSQQASVSQSQQASVSQSQQASVSQSQQSSNAYSAASNAASSVSQASSDSSYFNSQVVQSALSSSLQSSSALSSIAYGQTSANINDVAAAVARSVSQSLGVSQQAAQSVISQQLASAGSGASAQTLAQLISSAVSSLVQQSGTVSAGQEQSISQSLSSSILSSLSQVVAQRPLPVPAPRPLPAPLPAPRPIPAPLPRPVPI)_4_
**Chimeric Sequences (Host Organism and Construct)**
***E. coli* FlAS_3_ with and without C-term:**
(GPGGAGPGGAGPGGAGPGGAGPGGAGPGGAGPGGAGPGGAGPSGPGSAAAAAAAAGPGGAGPGGAGPGGAGPGGAGPGGAGPGGAGPGGAGPGGAGPGGAGPGGAGPSGPGSAAAAAAAAGPGGAGPGGAGPGGAGPGGAGPGGAGPGGAGPSGPGSAAAAAAAAGPGGAGPGGAGPGGAGPGGAGPGGAGPGGAGPGGAGPGGAGPGGAGPGGAGPGGAGPGGAGPSGPGSAAAAAAAAGPGGAGPGGAGPGGAGPGGAGPGGAGPGGAGPGGAGPGGAGPGGAGPGGAGPGGAGPGGAGPGGAGPGGAGPGGAGPGGAGPSGPGSAAAAAAAA)_3_
***E. coli* chimeric A_4_S_8_ with and without C-term:**
(GGAGPGGAGPGGAGPGGAGP4GGPSGPGSAAAAAAAAGP8)_8_

**Table 2:** Parr vessel solubilization of rMaSp2 with C-term at varying temperatures and pressures.

Temperature	Max Pressure	Time to Target Temperature/Max Pressure
120 °C	127 psi	16 min
130 °C	131 psi	16 min
135 °C	140 psi	17 min
140 °C	155 psi	20 min
150 °C	161 psi	25 min
